# “I struggle to count my blessings”: recovery after hip fracture from the patients’ perspective

**DOI:** 10.1186/s12877-018-0716-4

**Published:** 2018-01-19

**Authors:** Vigdis Bruun-Olsen, Astrid Bergland, Kristi Elisabeth Heiberg

**Affiliations:** 1 Department of Medical Research, Bærum Hospital, Vestre Viken Hospital Trust, Drammen, Norway; 2 OsloMet - Oslo Metropolitan University, Oslo, Norway

## Abstract

**Background:**

Recovery outlooks of physical functioning and quality of life after hip fracture have not changed significantly over the past 25 years. Previous research has mainly dealt with causalities and acute treatment, while the recovery process from the patients’ perspective has been less comprehensively described. Expanded knowledge of what the patients consider important in their recovery process may have important consequences for how these patients are treated in the future and thereby on future patient outcomes. The aim presently is therefore to explore how elderly patients with hip fracture enrolled in an ongoing RCT have experienced their recovery process.

**Method:**

The study was qualitative in design. Eight frail elderly in recovery after hip fracture (aged 69–91) were interviewed in their home four months after their fracture. The interviews covered issues related to their experiences of facilitators and barriers throughout the different stages in the recovery process. The patients were already enrolled in an ongoing randomized controlled trial, examining the effects of habitual functional training during their short term stays at nursing homes. The patients were chosen strategically according to age, gender, and participation in rehabilitation. The interviews were recorded, transcribed and subjected to a method of systematic text condensation inspired by Giorgi’s phenomenological method.

**Results:**

The results revealed that the patients’ experiences of the recovery process fell into three main themes: “Feeling vulnerable”, “A span between self-reliance and dependency” and “Disruption from a normal life”. The feeling of gloominess and vulnerability persisted throughout. Being in recovery was also experienced as a tension between self-reliance and dependency; a disrupted life where loss of mobility and the impact of age was profoundly present.

**Conclusion:**

Being in recovery after hip fracture was experienced as a life breaking event. Based on these findings, increased focus on individualized treatment to each patient through each stage of the recovery process should be emphasized.

## Background

Hip fracture is one of the most common fractures in older people. The annual incidence rate of hip fractures in Norway is approximately 9000 patients [1], 71% of them are women [2]. Worldwide, the burden of hip fractures is a serious health problem - for the patients as pain and functional decline, for their families, and for the society in a health economic perspective [3]. In general, both short- and long term recovery for these patients are poor, with permanently reduced physical functioning [4–6] and an increased 1-year mortality rate of 18–33% [4]. Despite extensive research and medical advances in the field of care after hip fracture, recovery outlooks of physical functioning and quality of life have not changed significantly in the past 25 years [7]. Previous research in the field has mainly dealt with the causalities and acute treatment, but the diversity of the recovery process from the patients’ perspective has been less comprehensively described [8]. Expanded knowledge of what the patients consider important in their recovery process may have important consequences for how these patients are treated in the future and thereby have an influence on patients’ outcomes. Therefore, further research on the recovery process from the patients’ perspective is needed.

There seem to be few studies focusing on the prolonged recovery process after hip fracture as experienced by the patients. The studies were either undertaken two to five days after the fracture [9], between hospital discharge and three months [10], at one month and four months [11], at one year after the fracture [12], or in community health care [13]. There seems to be scarce knowledge on the experiences of having a fracture and being in recovery throughout several phases of the recovery process [3, 14]. We assume that rehabilitation after hip fracture ought to contain functional training in walking and transfers. However, there are diverse rehabilitation strategies after hip fracture [15–17]. There do not seem to be one set of guidelines for hip fracture rehabilitation after discharge from hospital [18–20]. We have therefore initiated and started to enroll patients in a randomized controlled trial (RCT), the HIPFRAC study, to examine the effects of habitual functional training while the patients are at short-term stays in nursing homes before they are returning to home [21]. However, while standardized assessments capture valuable information on the patient’s physical and mental functioning, they may not capture information of relevance for the individual patients in their recovery process [22]. Given that patients are experts on own context, it is important to ensure that the patients are given the opportunity to convey how they experience what factors that have been important to them to be able to recover.

Moreover, it is increasingly expected that health care evaluation should include domains of health that are important to patients, captured by well-developed patient-reported outcome measures (PROMs). They aim to assess how patients experience their health condition or associated treatment [23]. For this patient group, we were unable to identify a PROM specific to the assessments of patients with hip fracture. Clarity with regard to the outcomes of health care that the patients consider important and relevant does not seem to exist [11, 24]. Developing patient-centered strategies, in which PROMs should be an important part, are therefore important for patients with hip fracture [3, 25]. Evidence illustrates that patient-centered care for individuals with multiple health problems can improve outcomes, facilitate connections between patients and health professionals, and make care more cost-efficient [25]. The scope of this article is therefore to explore the patients’ perspectives on their recovery process after hip fracture in greater depth.

By shedding light on the patients’ experiences; from hospital stay, during short term stay, and until the patients have returned to home, we may be able to identify the patients’ perceived challenges throughout a prolonged period of time. The patients’ perceived challenges may be of importance for future PROM-based assessments and also for future clinical decision making.

The aim of the present study is therefore to explore how elderly patients with a hip fracture have experienced their recovery process through the different stages between three to four months after the fracture.

## Method

### Research design

Presently, and to explore how elderly patients have experienced their recovery process after hip fracture, a phenomenological approach was chosen. Phenomenology is both considered as a philosophical approach and a research method which gives the possibility to obtain an understanding of the meaning of a given phenomenon [26]. Understanding the participants’ perspectives and experiences required an ability to openly meet the patients’ expressions. Our aim was to develop insights into how people with hip fracture gave meaning to factors that had hindered and facilitated their recovery process. In this research process, the researcher was considered as an active participant in the development of knowledge. In this way new questions were continuously raised, albeit they were not universal truths [27].

### Participants

The subjects initially participated in an ongoing randomized controlled study where the effects of habitual functional training, initiated by the research physiotherapist and performed by the nurses during short-term stays, were compared to usual care [21]. In the randomized study, a total of 160 patients are to be included. The patients were recruited after acute low-energy hip fracture surgery from a hospital nearby Oslo. Further inclusion criteria were an age of 65 years or older, home-dwelling prior to the fracture, and competent to give informed consent. Exclusion criteria were limitations to walk more than 10 m with or without a walking aid, a score on Minimal Mental Status Evaluation (MMS-E) [28] of less than 15 points in the acute phase, a pathological fracture, limited life expectancies, contraindications for training, or incapability to understand and speak Norwegian. All included patients had given written informed consent to participate in the study.

Presently, the subjects in this interview study were chosen among those who had completed their 3-month measurements in the ongoing RCT [21]. In line with the phenomenological approach, a strategic selection of individuals with different backgrounds, such as age, gender, and ability to express their experiences, was included. All patients who were invited to be interviewed accepted to participate. Thus a sample of eight subjects, six women and two men, aged from 69 to 91 years were included in the study (Table 1). The subjects were interviewed once; at three to four months after having a hip fracture, about their experiences with the different stages of the recovery process.

**Table 1:** Socio-demographics and clinical characteristics of the interviewed subjects (*n* = 8)

Subject	Age range	Gender	Educational level	Type of procedure	Short-term stay
1	85–89	F	> 12 y	4 hip screws and osteosyntesis	yes
2	65–69	F	≤ 12 y	Hemi prosthesis	yes
3	85–89	F	≤ 12 y	Hemi prosthesis	yes
4	70–74	M	> 12 y	Hemi prosthesis	no
5	75–79	M	> 12 y	Hemi prosthesis	yes
6	80–84	F	≤ 12 y	Hemi prosthesis	yes
7	90–94	F	> 12 y	4 hip screws and osteosyntesis	yes
8	70–74	F	> 12 y	4 hip screws and osteosyntesis	yes

### Data collection

The interviews were performed and audio recorded in the participants’ homes. We used semi- structured interviews with open-ended questions according to an interview guide.

In the beginning of the interviews, the subjects were asked about how they managed functionally before the fracture and their present functional status. Then we encouraged them to express and reflect about how they experienced their hospital stay with focus on facilitators and barriers related to rehabilitation, nutrition, safeguarding, and empathy from the hospital staff. Then they were asked about experienced facilitators and barriers during their short-term stay in the nursing homes (for those involved), and thereafter, how life in general was experienced at present. Examples of questions were: “Would you please tell me about your experiences with having a hip fracture?” “Would you please tell me more about what it meant to you?” “Please describe what you perceived as barriers or facilitators during hospital stay.” “Please describe your experience of participation in the rehabilitation intervention.” “Please describe what facilitated the recovery process after discharge from hospital.” Encouraging prompts were made throughout, such as “What do you think about this?”, or “Can you tell me more about this issue?” “Is there anything else you want to tell me, something you find important?” Follow-up questions were*:* “Could you please explain.. .?” “Could you tell me more about.. .?”

When conducting the interviews, the aim was to maintain an open, non-judgmental attitude. Emphasis was placed on listening to the responses of the participants. The participants were encouraged and given time to fully explain their experiences. Thereafter they were invited to reflect upon them. Our intention was to let each subject tell their own story, by fully describing recovering factors as well as barriers experienced.

VB-O and KEH carried out the interviews every second time, giving each other continuous feedback. Thus, the way in which the interviews were performed was quality assured. The interviewers were female physiotherapists and researchers, and they were not involved in the care of the participants at any stage in the recovery process. Each interview lasted from 40 to 60 min.

### Data analysis

The interviews were transcribed and the process of analysis started. Our aim was to generate knowledge on factors that were experienced as facilitators or barriers through the different stages of the recovery process. Thereby we wanted to stay close to the participants’ view of what had felt significant and important to them. The analysis was performed according to a method of systematic text condensation inspired by Giorgi’s phenomenological method [29], as modified by Malterud [30]. This method contained four steps: The first step was to familiarize and obtain an overall impression of the material. Presently, every interview was read several times independently by two of the authors. The second step was to identify qualitative meaning units directly related to the aim of the study. A meaning unit is a text fragment containing some information about the research question [30]**.** These meaning units were coded. Statements that dealt with experienced facilitators and barriers in the recovery process, and conceptions of what influenced recovery, were extracted from the interviews. Thereby a concentrated and representative version of the dialogue was achieved. The extracted meaning units were contrasted to each other to reveal similarities and differences. This coding resulted in groups of similar meaning units. The aim was to ensure that the groups did not overlap and that there was empirical support for each of them. The third step was to condense the contents of each coded group into subthemes. In step four, the various subthemes reflecting patterns and concepts of the participants’ experiences of facilitators and barriers in the recovery process were merged into overarching themes. There was a constant interplay in the entire process between the various steps of the analysis. Presently, three overarching themes containing several subthemes are presented in the Result section. An example of the analytic process is presented in Table 2.

**Table 2:** An example of the analytic process

Meaning units	Coded groups	Subthemes	Overarching theme
*“I felt like being in another world. Suddenly other people decided on what I should do, and I did precisely what they told me, I did not dare to do otherwise”.*	Other people decided over me	Feeling of subservience	Feeling vulnerable
*“I have been down in a black hole and I do not seem to get up again”.*	Being in a black hole	Feeling of gloominess and hopelessness	
*“A fracture like this…. and suddenly one starts to think about the future and how it is possible to manage at home. It is easy to get negative thoughts”.*	Negative thoughts about the future		

Two of the authors performed the interviews. Data gathering and analysis was performed until saturation was reached. After six interviews, no new categories reflecting the study aim could be developed from the data. We performed altogether eight interviews, while the last two interviews were analyzed without producing any additional changes in the structure. The same two authors also read and analyzed the text. In the analytic process a consensus was reached through continuous discussion between the authors.

Making the researchers’ preconceptions visible is a key aspect of qualitative research [30, 31]. At the time of the interviews and analysis, our immediate preconceptions were linked to the experiences of working and doing research among older people with hip fractures. As physiotherapists our focus in rehabilitation is on activity and training. We had a preconception that the patients also were concerned about the importance of activity to recover. Instead of bracketing these, the preconceptions enabled us to challenge some of the interviewees’ statements and descriptions and discuss them throughout the analytic process.

## Results

The patients’ experiences of the recovery process were shown to fall into three main themes: “Feeling vulnerable”, “A span between self-reliance and dependency”, and “Disrupted from a normal life”. Excerpts from the content of the participants’ interviews are presented in italics, and the quotation marks are referring to gender and age.

### Feeling vulnerable

The first main theme identified how the participants interpreted and experienced their own vulnerability and passivity as unexpected, especially during hospital stay, but also after returning to home. This theme emerged from the subthemes “Feeling of subservience” and **“**Feeling of gloominess and hopelessness”.

#### The feeling of subservience

Some of the participants described that they did not remember much from the days they spent in hospital. Time and again they expressed that during their hospital stay they felt totally cut off from the world. Moreover, they described the feeling of being in hospital as being submissive and a passive recipient more than an active part in their own recovery process*.* One of the women explained it in this way:

*“I felt like being in another world. Suddenly other people decided on what I should do, and I did precisely what they told me, I did not dare to do otherwise”.* F1

Quite a few of the patients experienced that during their hospital stay they were offered little opportunity to be actively involved in making decisions about their recovery. Experiences of having to adapt to routines and procedures entailed a feeling of passivity and having to do what the staff told them.

#### The feeling of gloominess and hopelessness

The explicit feeling of gloominess was mentioned time again by our participants throughout the different stages of the recovery process. This feeling came to light in various expressions. One of the participants expressed her persistent gloominess as a feeling of emptiness, a barrier, and a black hole:

*“After the hip fracture I have felt depressed for the first time in my life. I feel totally empty. And the gloominess persists even now (four months after the fracture). It is like having fallen into a black hole and being unable to get up again”.* F1

An old woman in the early nineties, who had recovered well despite her age, expressed that she felt gloomy; despite everybody tried to do what they could for her:

*“I do feel gloomy, yes….. I know that the people around me do what they can for me, but they can’t be me”. Although I have a lot of “blessings” in my life, I now struggle to “count my blessings”.* F7

Yet another participant expressed her feeling of vulnerability and fear of the future as a barrier for her own recovery:

*“Suddenly I feel old. I have never had such thoughts before. Suddenly one starts to count the years ….how many years do I have left”?* F7

Having a hip fracture seems to have changed their lives in an existential way.

### A span between self-reliance and dependency

This theme identified how the participants interpreted and experienced their recovery process as a span between own effort and other persons’ actions. This theme emerged from the following subthemes: “The gap between expectations and reality”, “Recovery as self-reliance”, and “Recovery: actions from others”.

#### The gap between expectations and reality

After discharge from hospital, the participants were transferred to either a short-term stay for further care and rehabilitation, or to home. At this stage in their recovery process, time and time again the participants seemed to slowly realize that there was a gap between how far they had expected to recover and how far they actually had recovered. This gap was experienced as a frustrating hurdle and barrier in their recovery process, reflected in the following statements:

“*My problem is that I expected this to take 14 days. I thought that after three weeks I would be able to walk without crutches. I had a friend who had a total hip arthroplasty and he walked without crutches after 14 days. I have been urgently waiting for my own functional recovery. Why is it taking such a long time? ….. It has been very frustrating. If I had known, it would have been easier for me…”* M4

The impatience and frustration of the patients regarding the gap between their expectations for own recovery and the reality they experienced were highlighted in various ways:

*“My expectations for my own recovery were much higher than reality, and that have made me frustrated and impatient”.* F3

*“I’m rather impatient as a person, so I would have liked to know how long it would take for me to be back to normal again ….. I’d expected more follow-up at the short-term stay”.* M5

Another participant expressed this gap differently:

“*The short-term stay was not as I expected. I expected to be in activity, doing things.* *I* *had to tell the physiotherapist that I needed a walking aid instead of a wheelchair, so I could go where I wanted. Before I was transferred to the short-term stay, they said I would get all the help I could think of … walking aids, seat raiser etc. However, when I got there, they did not help me much to be able to manage. I still regard myself as a younger person. With a little help and exercise, I would have managed to walk on my own”.* F2.

#### Recovery as self-reliance

Time and again the participants expressed that during the short-term stays their ability to recover was a lonely process mostly dependent on their own effort and self-reliance. One female patient vividly described how she felt that she had to manage everything by herself:

“*I went by myself to training and got out of the high walker entirely by myself,…….and I said to the nurse that now I have to start washing myself because I am going home. Yes, the nurse said, − without showing any interest. The short term stay felt disruptive”.* F1

Two other patients also experienced that recovering was more or less entirely up to them:

*“I had to set my own goals for recovery. It was all up to me. I myself had to learn to walk so I could manage stairs”.* F2.

*“What helped me was that I walked in the corridors with the high walker, by myself”.* M5

#### Recovery: Dependent on actions from others

Some participants clearly described that they believed that treatment and action from others influenced and thereby facilitated their recovery process. Lack of physiotherapy and activity during short term stays was expressed as a barrier and a hindrance, while encouragement was experienced as a facilitator:

“*Before I went to the short-term stay, I was promised physiotherapy three times a week. During my short-stay I only had physiotherapy three times in three weeks. I literally sat in a wheelchair for three weeks”.* F2

The participants stressed the importance of the professional and personal manner of the health care workers to enable them to recover from a vulnerable situation. Recognition from others was perceived as a positive and encouraging experience. One of them described the recognition from one of the nurses:

*“One nurse was nice because she saw me ….. she noticed each of us. That is the essence. One cannot treat two different patients in the same way”.* F1

One of the other participants expressed that the positive encouragement from the physiotherapist had been of vital importance:

“*The physiotherapist, and the way she treated and encouraged me and explained things, has been vital for my recovery*”. M4

Another participant also recognized that trust and reliance on the health care workers was perceived as a sort of facilitator in her own recovery process:

“*In hospital I had to pull myself together and do what I was told. I understood that I had to listen to what the health care workers said to recover fast. And I received their help with gratitude”.* F3

“*During hospital stay they encouraged me to walk to the bathroom. The encouragement was important; otherwise it had been easy to lie quietly in bed and do nothing, because of the pain……”* F2

### Disrupted from a normal life

This theme identifies how the patients experienced the consequences of the fracture four months after the incidence as a disruption from a normal life. Many of the participants described life as different from before. They feared they would never regain their former life. This theme consists of the following subthemes: “Less independence and mobility” and “The impact of age”.

#### Less independence and mobility

One of the participants described the time before and after the fracture as a personal transformation, from being an independent person to one who others believed was in need of surveillance. She experiences herself as a totally different person:

*“After the fracture, my children decided that I had to sell the house and move out. Naturally, that was necessary …... Everything was well before the fracture. After the fracture the consequences was a disrupted life, I have to say…..suddenly I was under surveillance”.* F3

One of the younger participants expressed this transition in a different way. She expressed that even after four months she felt bodily different:

*“My body feels very cold, very warm, very, eh…different. Maybe I am trying too hard to handle this…. to heal.”* F8

#### The impact of age

Despite their age, after the fracture the patients suddenly seemed to realize the impact of age. Many of the participants did not believe they would be able to return to their former life. One youthful male patient described it like this:

*“I do not think I will return to my former life. I realize that… … earlier everything was ok”.* M4

Another participant expressed that even after four months the hip fracture had changed her attitude and hopes for the future. After the fracture she suddenly realized the impact of age and what she had lost.

*“I will never be the person I was before the fracture. I used to be in good shape, despite my age. Now I ask myself: What is there really to look forward to when you are ninety?”* F7

## Discussion

This study has explored the patients’ experiences through the different stages of the recovery process after hip fracture. The findings highlight that being in recovery from a hip fracture can be a long lasting endeavor, which may involve the feeling of vulnerability, a span between self-reliance and dependency, and later on - the feeling of a disrupted life. Thus, for many patients, a fractured hip is experienced as a life breaking event, which implies an uncertainty about the future and what would best support their recovery process.

In Norway, people sustaining a hip fracture have a mean age of 80 years and most often hip fractures occur in women [1, 2], which correspond well with the participants in our study. During hospital stay some of the participants felt vulnerable and cut off from the world. For some, this was experienced as a hindrance in their early recovery process. This is in line with findings from a newly published study [9] where ward culture during hospital stay may affect the recovery of the patient. To highlight how the patients experience the hospital culture as a hindrance or a facilitator for their recovery may be of importance, in order to better comply with the patients while they are in hospital, and thereby empower and strengthen their coping strategies to influence their recovery process.

In the mentioned study [9], the patients felt insecure and vulnerable during their first few days in hospital. In our study, the feeling of vulnerability and gloominess seemed to persist also after the patients had returned to home. The feeling of suddenly being depressed after going through a hip fracture is not uncommon for patients after such a health event [32]. In other studies older people have also reported that their lives were changed physically, personally, and socially, and that they struggle hard to take control of their future life [10, 33]. These are important findings that may lead to consequences regarding how these patients are to be treated by health care workers.

After discharge from hospital, the participants experienced that there was a definite gap between the expectations they had for their own recovery and the reality they met. One dominant conception among the subjects was that recovery was dependent on personal factors such as mobilizing your own will, motivation, and engagement in exercise. Similar findings were reported in a previous study [34]. Staying active and engaging in balance and strength exercises seems to be one of the most effective postoperative measures for the patients’ ability to return to their pre-fracture level and to regain independent ambulation. Some of the interviewees’ conceptions of recovery could be associated with psychological aspects, such as self-efficacy [35] and locus of control [36]. It has previously been reported that low self-efficacy, i.e. perceived ability to carry out an action, is associated with depression and anxiety [37–39] and closely connected to activity restrictions [40]. A study by Shaw [36] found that a strong internal locus of control can be associated with less physical disability one month after the fracture. It is a common view that people who have a belief that they have control over their own health are more likely to participate in activities and comply with treatment [8]. Traces of both internal- and external locus of control are found in our data. Based on these findings, implications for future assessments of recovery after hip fracture should include PROMs assessments with the patients’ self-efficacy and locus of control included, to better target the patient’s ability to adhere and comply with treatment.

Nurses, physiotherapists, and medical doctors are traditionally influenced by the idea of a health care worker as a person whose primary task is to inform the patient of what to do to achieve better health [41]. The patient should then follow the advice. Until recently, most literature refers to this as compliance or adherence. A new construct is now introduced as concordance. This reflects the contemporary view of the patients as collaborators in their own recovery process [42]. The patient accomplishes functional improvements and attains his or her own goals, as developed through a patient and health care worker partnership. The findings from the present study, in which some of the participants described a feeling of passivity, accentuate research reporting that the negative consequences of a hip fracture are substantial and long lasting. These results may give implications for future organization of health care. Considering the remaining long lasting consequences of the fracture reported here as well as in several other studies [33, 43, 44], it is our opinion that extra effort should be made to include the patient as a collaborator. Thereby, the patient’s self-reliance and hope for recuperation may be supported. This may enhance patient outcomes.

### Methodological reflections

This study has its strengths and weaknesses. The aim of our study was to explore the experiences of the recovery process among some of the patients who participated in an ongoing RCT, to expand on the knowledge of their recovery. The design is qualitative, which implies that the study should be assessed by the means of trustworthiness, which comprises credibility, transferability, confirmability, and dependability [27]. Credibility relates to whether the findings are “true” and are based on faithful descriptions. The present conditions for data collections, interviews, sampling, and how well the data are covered in the main categories are important aspects to consider. Both men and women with varying age and civil status were included in the sample and contributed to sample variation. Two of the authors participated in the process of analysis, which further increases credibility and confirmability. In addition, the analytic process was made transparent in Table 2, and by the use of quotations in the presentation of results. Moreover, the interviews were performed in the participants’ homes, which may have strengthened the method, since home environment should be seen as a safe place in which to talk freely. Some interviews were shorter than others, but all of them lasted from 40 to 60 min. The descriptions of the text were considered to be overall detailed and rich. According to the criteria for reporting qualitative research (COREQ) [45], we have as researchers clarified our credentials and occupations, gender, and former experience to further improve credibility of the findings. Thus, the overall findings in the study are viewed as credible. Dependability relates to what extent the findings are consistent and can be repeated. Data were collected using a semi-structured interview guide. The guide ensured that the interviewees were asked questions within the same areas. Moreover, some of the results from the study were supported by similar findings from studies on patients’ perspectives on recovery one year after hip fracture where the feeling of anxiety and gloominess seems to persist as long as one year after discharge [3, 8]. Therefore, the patients’ expressions of depression in the present study were recognizable. We have attempted to describe the research process as accurately as possible and followed the steps in the analysis. We have used quotations to illustrate the subthemes and thereby ensuring concordance between the interviews and the themes. The fact that more than one researcher conducted the interviews and performed the analysis can be seen as enhancing the credibility and dependability of the findings.

One weakness may be that we have performed interviews only once between three and four months, asking the patients to recall their experiences from the days at the hospital, from the short-term stay in a nursing home, and from the period in their own home. The recovery process would possibly have been more fully described if we had repeated the interviews later on, or performed the interviews at different time points. On the other hand, in a study by Griffith [11] there was planned a follow-up interview at twelve months after hip fracture. These interviews did not take place due to the participants being old and the apparent problem with recall bias.

### Clinical implications

A strong internal locus of control can be associated with adherence and better outcomes in physical activity one month after hip fracture [36]. The findings from a study by Shaw [36] suggest that health care interventions that enhance perceived internal control by patients during rehabilitation, may result in better physical outcomes. Clinical implications would be that approaches and activities supporting patients’ self-reliance should be given priority to enable the patients to comply with treatment. Based on former studies and the present findings, one may suggest that it is desirable to support the patients on an individually optimal level through their recovery process to enhance outcomes. The interventions should also be individually adjusted to each patient’s needs and personal prerequisites to enhance adherence to the intervention and thereby possibly improve outcomes.

## Conclusion

The findings from this study highlight that being in recovery from a hip fracture is a long lasting endeavor which may involve an unexpected feeling of vulnerability, and that recovery is perceived both as dependent on self-reliance and on external factors outside oneself. All in all, being in recovery is seen as a break from a normal life. The knowledge may help us to understand how recuperation after hip fracture is perceived. A broken hip may be seen as a minor event by health care workers, but is experienced as a disruptive event for many patients. Based on these findings, increased focus on individualized treatment and care to each patient throughout each stage in the recovery process should be emphasized.

## Abbreviations

COREQ: Criteria for reporting qualitative research
PROMs: Patient reported outcome measures
RCT: Randomized controlled trial
